# Growth Factor Concentrations in Human Milk Are Associated With Infant Weight and BMI From Birth to 5 Years

**DOI:** 10.3389/fnut.2020.00110

**Published:** 2020-07-29

**Authors:** Laura Galante, Shikha Pundir, Hanna Lagström, Samuli Rautava, Clare Marie Reynolds, Amber Marie Milan, David Cameron-Smith, Mark Hedley Vickers

**Affiliations:** ^1^The Liggins Institute, University of Auckland, Auckland, New Zealand; ^2^Department of Public Health, University of Turku and Turku University Hospital, Turku, Finland; ^3^Centre for Population Health Research, University of Turku, Turku, Finland; ^4^Department of Pediatrics, University of Turku and Turku University Hospital, Turku, Finland; ^5^Department of Pediatrics, University of Helsinki and Children's Hospital, Helsinki University Hospital, Helsinki, Finland; ^6^AgResearch Limited, Grasslands Research Centre, Palmerston North, New Zealand; ^7^Singapore Institute for Clinical Sciences, Agency for Science, Technology and Research, Singapore, Singapore

**Keywords:** BMI, cGP, human breastmilk bioactives, IGF-1, growth factors, adiponectin, leptin, infant growth

## Abstract

**Background:** Human milk bioactives may play a role in infant health and development. Although the variability in their concentrations in milk is well-established, the impact of differential milk profiles on infant growth outcomes remains unclear. Thus, the aim of the present study was to investigate whether different concentrations of metabolic hormones are associated with different weight and BMI in infants beyond the first year of life.

**Methods:** Milk samples at 2.6 (±0.4) months after birth and anthropometric measures at 13 months, 2, 3, and 5 years were collected as part of the Finnish STEPS cohort study from 501 mothers and the respective 507 infants. Leptin, adiponectin, insulin-like growth factor (IGF)-1 and cyclic glycine-proline (cGP) in milk were analyzed. Multiple regression models and a repeated measures mixed model were used to examine associations between milk hormone concentrations and weight and BMI z-scores across time, at each time-point, and weight gain from birth to each follow-up visit. All models were corrected for birth weight, infant sex, duration of exclusive and total breastfeeding, time of introduction of solid foods and maternal pre-pregnancy BMI.

**Results:** Higher milk IGF-1 was associated with higher weight at 13 months (*p* = 0.004) but lower weight at 3 (*p* = 0.011) and 5 years of age (*p* = 0.049). Higher cGP was associated with lower weight across the 5 years (*p* = 0.019) but with higher BMI at 5 years (*p* = 0.021). Leptin and adiponectin did not display associations with infant growth at this time. Sex interactions were also absent.

**Conclusions:** Our results suggest that the interplay between human milk-borne IGF-1 and cGP is similar to that reported in other mammals and may have an important role in defining infant growth trajectories beyond the first year of life. Further research should explore the determinants and origins of these milk-borne compounds and evaluate their effect on infant growth and metabolism.

## Introduction

Breastfeeding is the gold standard for infant nutrition. However, the importance of human milk (HM) for infants goes beyond meeting their nutritional needs. It is well-established that HM consumption is associated with lower risk of longer-term morbidities, including obesity (1, 2) and metabolic syndrome (3), which constitute major public health issues worldwide (4). As the concept of nutritional programming during “the first 1,000 days of life” is gaining recognition (5, 6), HM-borne hormones that regulate metabolic function are seen as potential programming factors (7–9). Evidence from animal models suggests that HM leptin (10) influences infant eating behavior and body composition (11) and may underpin the protective effects of breastfeeding against obesity (12). The presence of adipokine receptors in the epithelial cells of the infant gastrointestinal tract (13, 14) together with the knowledge that human neonates are capable of absorbing intact macromolecules during the early neonatal period (15, 16) reinforces the hypothesis that these HM-borne molecules have a functional role in infant metabolism. Additionally, other hormones, including insulin-like growth factor (IGF)-1, together with its binding proteins and regulatory metabolites, including cyclic Glycine-Proline (cGP), also have a potential role in programming infant growth trajectories due to their well-established roles in linear growth, and body composition (17).

As variations in circulating adipokines (18), IGF-1 and cGP (19, 20) have been linked to various health and disease outcomes and significant differences in HM hormone concentrations have been described across mother-infant dyads (21–23), research has focused on associations between hormone concentrations in HM and infant growth outcomes (24–36). However, while some studies suggest that individual hormonal signatures in HM are associated with different infant growth trajectories, the findings of research in this area remains conflicting (37). Yet, the pattern of early postnatal growth beyond the first year of life is a known predictor of later metabolic health (38) and previous studies have shown that the composition of milk received by the infant plays an important role in determining growth trajectories (39).

In a recent study we reported on the association between HM concentrations of adiponectin, leptin, IGF-1 and cGP with maternal-infant characteristics in the STEPS cohort (40). We identified significant differences in the concentrations of HM-borne total protein, IGF-1, leptin and adiponectin across mother-infant dyads, particularly in association with maternal/perinatal/infant characteristics including pre-pregnancy BMI, maternal education, birth mode, gestational diabetes (GDM), and infant sex. Following our initial observations, in the present study we hypothesized that differences in HM composition would correlate with different weight and BMI z-scores in the infants from birth to 5 years of age.

## Materials and Methods

### Study Design and Population Characteristics

This study utilizes HM samples obtained from 501 mothers and 507 children participating in the Finnish longitudinal cohort, Steps to healthy development of Children (the STEPS Study) between 2008 and 2010, as described previously (40). HM samples from consenting mothers were collected 2.6 ± 0.4 months after birth by manual expression in the morning, from a single breast, as detailed previously (41). Study visit retention decreased from birth (*N* = 507) to 5 years (*N* = 391) but remained high at almost 80%. Maternal pre-pregnancy BMI was calculated from self-reported height and weight and clustered into the following categories: underweight < 18.5 kg/m^2^, normal weight 18.5–24.9 kg/m^2^, overweight 25–29.9 kg/m^2^, obese >29.9 kg/m^2^. Information regarding infant sex, birth weight and length were obtained from the Longitudinal Census Files. Information on feeding practices was collected through follow-up diaries completed by mothers (42). Exclusive breastfeeding was defined as the infant not receiving anything other than HM, with the exception of water, supplements or medicines. Total breastfeeding was defined as the infant receiving HM and any other liquid or food. Infant anthropometric measures (weight, length/height) were taken during follow-up visits at 13 months, 2, 3, and 5 years of age as previously detailed (42). These measurements were used to calculate the BMI and respective z-scores using references specific to the Finnish population (43). Weight gain was calculated as difference between each clinical visit and birth weight. The study protocol was approved by the Ethics Committee of the Hospital District of Southwest Finland in February 2007 and March 2015. Written informed consent was obtained from all participants.

### HM Analysis

HM analysis was undertaken in 2018 as described previously (40). In brief, leptin, adiponectin and IGF-1 in HM were analyzed using commercially available ELISA kits (human sensitive leptin ELISA, human adiponectin ELISA, human IGF-1 ELISA, Mediagnost, Germany), while cGP was analyzed via liquid chromatography tandem mass spectrometry (LC-MS). HM total protein was quantified by infrared spectrometry using the Direct Detect® technology (Merck, Germany) in order to normalize hormone concentrations. The intra- and inter-assay coefficients of variation, respectively, for the ELISAs (QCs supplied) were adiponectin (5, 6%), IGF-1 (3, 9%), and leptin (4, 8%).

### Statistical Analyses

For the purpose of this study we only used weight and BMI z-scores and weight gain as outcome variables, since these are the primary predictors for childhood obesity (44). The power provided by the smallest sample size (*n* = 391) amongst the follow-up visits was above 90% for multiple linear regression analyses at 5% significance level for the detection of small effect sizes (G^*^Power 3.1.9.2). Similar to our previous study, HM concentrations of leptin adiponectin, IGF-1 and cGP were corrected for total protein concentration in each sample (mg/ml) and reported as ng/mg of protein per ml (ng/mg). Outcome variables were assessed for normality with the Shapiro Wilk test. Since the distribution of weight gain was not normal, all weight gain variables were log10 transformed. Associations between adipokine and growth factor concentrations in HM and weight and BMI z-scores from 13 months to 5 years were analyzed using multiple linear regression models (to assess associations between HM composition and individual infant outcomes at single time-points) and repeated measures mixed models (to assess associations between HM composition and individual infant outcomes across time-points from 13 months to 5 years of age). Associations between hormone concentrations in HM and weight gain from birth to each clinical visit (13 months, 2, 3, and 5 years) was also analyzed through multiple linear regression models. Interactions of HM composition with infant sex were examined in each model. Maternal BMI class before pregnancy, birth weight, total duration of breastfeeding as well as the time of introduction of solid foods were used as correcting factors (45, 46) (fixed factors) in each regression model (both multiple linear regression and repeated measures), as they were significantly associated with infant growth outcomes which was the dependent variable in our cohort (weight z-scores, BMI z-scores and weight gain) during simple regression analysis. Gestational age and birth-mode were also considered as co-factors. However, since both variables strongly correlated with birth weight, we only used the latter (47). No random effects were evaluated in this study, but the use of repeated measures mixed models was adopted in order to appropriately deal with the missing data (e.g., any dropouts between clinical visits). All statistical analyses were performed using IBM SPSS (version 25).

## Results

### Population Characteristics

Of the 507 infants whose mothers had consented for HM hormone and growth factor analysis, 391 (77%) were followed up until 5 years of age.

The overall characteristics of the study population are reported in Table 1. Although the number of infants decreased from one visit to the other, the proportion in infant sex, and maternal pre-pregnancy BMI remained consistent throughout the study. As shown by the mean z-scores for weight and BMI, our study population did not differ from the general Finnish population.

**Table 1:** Characteristics for the study population at each clinical visit.

**Population characteristics**	**Birth**	**13 months**	**2 years**	**3 years**	**5 years**
**Anthropometry, Mean (SD)**					
*N* (%)	507 (100)	504 (99)	448 (88)	405 (80)	391 (77)
Missing (%)	0	3 (1)	59 (12)	102 (20)	116 (23)
Weight (kg)	3.490 (0.506)	10.352 (1.108)	12.941 (1.351)	15.260 (1.613)	19.987 (2.521)
Weight (z-score)	0.18 (0.889)	−0.19 (1.197)	0.08 (0.964)	0.09 (0.938)	0.22 (0.901)
BMI (kg/m^2^)	NA	NA	16.5 (1.2)	16.2 (1.1)	16.0 (1.3)
BMI (z-score)	NA	NA	0.04 (1.048)	0.21 (0.958)	0.08 (0.897)
Weight gain (kg)	NA	6.86 (1.05)	9.44 (1.31)	11.76 (1.52)	16.49 (2.43)
**Infant sex**, ***N*** **(%)**					
Male	273 (54)	270 (54)	245 (55)	219 (54)	212 (54)
Female	234 (46)	234 (46)	203 (45)	186 (46)	179 (46)
**Maternal pre-pregnancy BMI**, ***N*** **(%)**					
Underweight	20 (4)	19 (4)	20 (4)	19 (5)	16 (4)
Normal weight	343 (68)	343 (68)	302 (67)	281 (69)	277 (71)
Overweight	91 (18)	91 (18)	85 (19)	70 (17)	63 (16)
Obese	51 (10)	51 (10)	41 (9)	35 (9)	35 (9)

### HM Adipokine and Growth Factor Composition and Infant Growth

As shown in Table 2, higher concentrations of HM IGF-1 were associated with higher weight at 13 months and lower weight at 3 and 5 years, while higher milk-borne cGP was associated with lower weight at 13 months and overall with lower weight z-scores across the 5 years, as indicated by the results of the repeated measures regression (Table 2). Higher IGF-1 was also associated with lower weight gain from birth to 2 years (Table 3) and higher cGP was associated with higher BMI at 5 years of age. No significant associations were found between HM leptin and adiponectin and infant growth and there were no sex-specific interactions (data not shown).

**Table 2:** Associations between weight z-scores at each follow-up visit and over time and HM composition.

**Weight z-scores**										
	**Weight 13 months**		**Weight 2 years**		**Weight 3 years**		**Weight 5 years**		**Weight z-score across time**	
**HM composition**	**B (95% CI)**	***p***	**B (95% CI)**	***p***	**B (95% CI)**	***p***	**B (95% CI)**	***P***	**B (95% CI)**	***p***
Leptin (ng/mg)	0.019 (−0.058, 0.096)	0.628	−0.019 (−2.018, 1.980)	0.985	0.088 (−2.172, 2.348)	0.939	0.227 (−1.917, 2.372)	0.835	0.020 (−0.062, 0.103)	0.623
Adiponectin (ng/mg)	0 (−0.017, 0.018)	0.955	0.236 (−0.169, 0.640)	0.245	0.304 (−0.124, 0.732)	0.164	0.242 (−0.171, 0.655)	0.25	0.108 (−0.178, 0.395)	0.745
IGF-1 (ng/mg)	**0.033 (0.011, 0.056)**	**0.004**	–0.367 (−1.020, 0.286)	0.27	–**0.961 (**–**1.705, 0.218)**	**0.011**	–**0.742 (**–**1.471**, –**0.014)**	**0.046**	0.026 (−0.013, 0.065)	0.187
cGP (ng/mg)	–**0.024 (**–**0.042**, –**0.006)**	**0.009**	−0.324 (−0.790, 0.143)	0.174	0.075 (−0.327, 0.134)	0.714	0.304 (0.784, 1.538)	0.215	–**0.023 (**–**0.042**, –**0.004)**	**0.019**
IGF-1/cGP ratio	**0.006 (0.001, 0.012)**	**0.018**	0.035 (−0.128, 0.199)	0.674	−0.109 (−0.228, 0.011)	0.075	−0.097 (−0.228, 0.034)	0.146	0.005 (−0.005, 0.015)	0.292
Protein (mg/ml)	3.817 ^*^ 10^−05^ (−0.002, 0.002)	0.962	−0.017 (−0.059, 0.024)	0.414	−0.016 (−0.058, 0.541)	0.462	−0.022 (−0.060, 0.017)	0.265	0 (−0.002, 0.002)	0.883

*Values represent parameter estimates obtained by each individual fitted regression model with the following cofactors: maternal pre-pregnancy BMI, birth weight, infant sex, total breastfeeding duration and introduction of solid foods. IGF-1, Insulin-like growth factor-1; cGP, cyclic Glycine Proline; B, unstandardized beta coefficients. BMI and weight z-scores were calculated using reference values specific to the Finnish population (43). Weight gain was calculated as difference between each clinical visit and birth weight. Bold values indicate significant p-values*.

**Table 3:** Associations between weight gain and BMI z-scores and HM composition.

	**Δ Weight from birth [log10(Kg)]**							
	**Weight gain from birth to 13 months**		**Weight gain from birth to 2 years**		**Weight gain from birth to 3 years**		**Weight gain from birth to 5 years**	
**HM composition**	**B (95% CI)**	***p***	**B (95% CI)**	***p***	**B (95% CI)**	***p***	**B (95% CI)**	***p***
Leptin (ng/mg)	−0.008 (−0.142, 0.125)	0.905	0 (−0.146, 0.145)	0.995	0.012 (−0.131, 0.155)	0.872	0.073 (−0.087, 0.232)	0.371
Adiponectin (ng/mg)	0.016 (−0.011, 0.043)	0.256	0.018 (−0.009, 0.045)	0.193	0.014 (−0.012, 0.040)	0.281	0.002 (−0.027, 0.031)	0.882
IGF-1 (ng/mg)	−0.023 (−0.068, 0.021)	0.305	–**0.060 (**–**0.107**, –**0.014)**	**0.011**	−0.047 (−0.094, 0)	0.051	−0.050 (−0.106, 0.006)	0.083
cGP (ng/mg)	−0.022 (−0.055, 0.010)	0.177	0.005 (−0.021, 0.030)	0.73	0.019 (−0.012, 0.050)	0.228	0.009 (−0.028, 0.047)	0.627
IGF-1:cGP ratio	0.002 (−0.009, 0.013)	0.667	−0.007 (−0.014, 0.001)	0.075	−0.006 (−0.015, 0.002)	0.148	−0.009 (−0.019, 0.002)	0.102
Protein (mg/ml)	−0.001 (−0.004, 0.002)	0.428	−0.001 (−0.004, 0.002)	0.435	−0.001 (−0.004, 0.001)	0.243	−0.003 (−0.006, 0)	0.086
	**BMI z-scores**							
	**BMI 2 years**		**BMI 3 years**		**BMI 5 years**		**BMI across time**	
**HM composition**	**B (95% CI)**	***p***	**B (95% CI)**	***p***	**B (95% CI)**	***p***	**B (95% CI)**	***p***
Leptin (ng/mg)	0.522 (−1.898, 2.942)	0.672	−0.643 (−2.675, 1.390)	0.535	0.067 (−2.0.40, 2.175)	0.95	0.059 (−1.477, 1.596)	0.939
Adiponectin (ng/mg)	0.039 (−0.435, 0.513)	0.871	0.348 (−0.053, 0.749)	0.089	0.089 (−0.315, 0.492)	0.666	0.104 (−0.235, 0.442)	0.548
IGF-1 (ng/mg)	−0.818 (−1.755, 0.119)	0.087	−0.542 (−1.336, 0.252)	0.181	−0.480 (−1.270, 0.309)	0.233	−0.615 (−1.301, 0.070)	0.078
cGP (ng/mg)	−0.213 (−0.813, 0.387)	0.486	0.276 (−0.144, 0.696)	0.197	**0.507 (0.076, 0.938)**	**0.021**	0.149 (−0.202, 0.500)	0.405
IGF-1:cGP ratio	0.020 (−0.191, 0.232)	0.851	–**0.212 (**–**0.312**, –**0.113)**	***p*** **< 0.001**	–**0.235 (**–**0.350**, –**0.120)**	***p*** **< 0.001**	−0.130 (−0.300, 0.040)	0.133
Protein (mg/ml)	−0.015 (−0.067, 0.038)	0.583	−0.010 (−0.056, 0.035)	0.656	−0.017 (−0.061, 0.026)	0.435	−0.013 (−0.047, 0.020)	0.436

*Values represent parameter estimates obtained by each individual fitted regression model with the following cofactors: maternal pre-pregnancy BMI, birthweight, infant sex, total breastfeeding duration and introduction of solid foods. IGF-1, Insulin-like growth factor-1; cGP, cyclic Glycine Proline; B, unstandardized beta coefficients. BMI and weight z-scores were calculated using reference values specific to the Finnish population (43). Weight gain was calculated as difference between each clinical visit and birth weight. Bold values indicate significant p-values*.

## Discussion

The present study shows that HM bioactive concentrations 3 months after birth were significantly associated with infant growth, even when adjusted for important confounding factors including birth weight, maternal pre-pregnancy BMI, total duration of breastfeeding and timing of introduction of solid foods. Our results suggest that milk-borne IGF-1 and cGP are related to weight and z-scores up to 5 years of age and weight gain up to 2 years of age.

While IGF-1 is the main growth factor during infancy (48), results on the relationship between different concentrations of IGF-1 in HM and infant growth outcomes are conflicting (49, 50). In the current cohort, concentrations of IGF-1 in HM collected 3 months after birth were associated with higher weight z-scores at 13 months and associated with lower weight z-scores at 3 and 5 years of age. Previous work by Ong et al. reported that circulating concentrations of IGF-1 at 5 years of age were inversely correlated to birth weight and positively correlated to current weight and weight gain from birth to 2 years of age, concluding that circulating IGF-1 in childhood is linked to growth rates in infancy (51). In the present study we found that HM IGF-1 concentrations were significantly associated with the same outcomes (i.e., weight, weight gain) but in the opposite direction compared to serum IGF-1. This suggests that HM IGF-1 could be involved in production pathways for endogenous IGF-1.

In this context, previous studies in both experimental animal models and clinical settings have reported that circulating IGF-1 during infancy is strongly regulated by nutrition (52, 53). In particular, breastfed infants have lower circulating IGF-1 and lower weight gain compared to formula fed infants (53). The only study to our knowledge that has examined IGF-1 concentrations in infant formula reported undetectable amounts of bovine IGF-1 [identical to human IGF-1 (54)] in the milk used in the formula production (55). While we do not know if bovine IGF-1 would be active in human infants or not (56), the presence of IGF-1 in HM as opposed to its absence/inactivity in infant formula might contribute to differences in endogenous IGF-1 regulation between breastfed and formula-fed infants.

While the impact of dietary IGF-1 on endogenous IGF-1 synthesis is not known, a previous study in a murine IGF-1R knock out model suggested an effect of IGF-1 signaling in adipocytes on systemic concentrations of IGF-1 (57). Such effects on systemic concentrations of IGF-1 was independent of alterations in growth hormone (GH) secretion and resulted in changes in somatic growth. Interestingly, decreased IGF-1 signaling resulted in higher weight gain and adipocyte hypertrophy. The study also found that increased IGF-1 signaling exerted a negative feedback on circulating IGF-1 (57). In light of this, we speculate that dietary and HM IGF-1 during early life might play a role in adipose tissue IGF-1 signaling, so that higher HM concentrations of IGF-1 may downregulate circulating IGF-1 and reduce infant weight gain and weight z-scores in the long term. Although in the present study we did not have the means to examine circulating concentrations of IGF-1 in the infants, it is possible that HM IGF-1 may explain why breastfed infants display lower circulating IGF-1 concentrations as compared to formula-fed infants. Considering that weight gain during infancy is a predictor of adiposity later in life (38) further studies investigating the role of HM IGF-1 as a mediator of weight gain during infancy are required.

While we did not measure IGF-1 binding proteins in our cohort, we analyzed cGP, a metabolite of IGF-1. cGP has been reported to play a regulatory function in relation to IGF-1 activity by maintaining IGF-1 homeostasis through competing against IGFBP-3 binding sites on IGF-1 (58). In our study, the analysis of the associations between weight and BMI z-scores and cGP concentrations in HM highlights a reversed action of cGP compared to that observed for IGF-1. This, together with the fact that both cGP and IGF-1 concentrations in HM were significantly associated with infant growth, further suggests that cGP may be modulating IGF-1 bioactivity in humans, as reported previously, with the ratio between the two indicating IGF-1 bioactivity (58). In this context, a higher ratio between IGF-1 and cGP (IGF-1/cGP), was associated with BMI at 3 and 5 years of age. Lastly, higher HM cGP concentrations were associated with lower weight z-scores trajectories over time suggesting that cGP may exert regulatory effects on IGF-1 activity in both directions (i.e., cGP may enhance the activity of IGF-1 when at low concentrations and reducing activity when IGF-1 is in excess).

Neither leptin nor adiponectin displayed significant associations with infant growth outcomes in the long term, despite some studies suggesting that these compounds may also have a role in infant development and growth (28, 59, 60). Overall, the existing literature in this context is conflicting [see (37) for review] and disparities in the findings are, in part, likely due to different approaches as regards sample collection and storage, laboratory approaches used and statistical analysis of the data, as infant growth is influenced by a complex array of factors. While some of these, including basic characteristics, that can be identified and controlled for, other factors, including potential synergism/antagonism between our hormones of interest and other bioactive factors in HM (37), are difficult to control for. The lack of associations in the present cohort between HM adipokine concentrations and infant BMI and weight does not preclude associations with infant body composition *per se*, as leptin in particular has been linked to eating behavior and fat mass (11).

The main strength of the present study was the large sample size and the prolonged follow-up data that allowed us to investigate associations between HM composition around 3 months postpartum and infant growth trajectories up to 5 years of age. Unfortunately, due to the lack of infant blood samples, we could not investigate the relationship between maternal HM bioactive concentrations and the respective circulating concentrations in the infant. Furthermore, while we were able to detect the concentrations in the samples given, the actual amount of bioactive IGF-1 received by the infant (i.e., not bound to binding proteins that limit its bioavailability) remains unknown. Certainly, the collection of maternal and infant blood samples as well as that of HM at more than one time-point would have aided in the formulation of hypothesis around possible mechanisms underlying our observations. While only one HM sample per mother was available, this was representative of mature milk, which is fairly stable across lactation (61), thus ideally representative of the entire lactation period from the first month to weaning. Of note, despite HM collection occurring on average 3 months after birth, we did not find any association between hormone concentration and exact time of collection, indicating that hormone concentrations were stable across slightly different collection times. The interpretation of the current findings are also limited by a number of factors (e.g., infant diet and physical activity) that were not available to us and that will impact on growth trajectories up to 5 years. Evaluating the eating behavior and body composition of the infant would also been valuable in relation to HM leptin.

Overall, although the variation in BMI and weight z-scores in the present population was generally small with values proximal to zero, IGF-1, and cGP displayed significant associations with infant growth outcomes. This, together with evidence arising from other studies in the same cohort (62), suggests a role for HM-borne compounds in contributing to postnatal growth trajectories. As the role of IGF-1 in association with adiposity in infancy remains poorly understood and current data remain conflicting, more comprehensive studies are required to investigate the mechanistic role of IGF-1 and cGP in HM and early development.

## Data Availability Statement

The raw data supporting the conclusions of this article will be made available by the authors, without undue reservation.

## Ethics Statement

The studies involving human participants were reviewed and approved by The Ethics Committee of the Hospital District of Southwest Finland in February 2007 and March 2015. The patients/participants provided their written informed consent to participate in this study.

## Author Contributions

LG designed the research questions, carried out laboratory and statistical analysis, interpreted the results, and drafted the manuscript. HL designed sample and data collection, assisted with statistical analysis, and contributed to the manuscript development. AM and CR helped design the research question and perform assay validations, and together with DC-S and SR edited the final manuscript. MV and SP supervised the entire process from laboratory analysis to submission of the manuscript. All authors have read and agreed to the published version of the manuscript.

## Conflict of Interest

AM is employed by AgResearch Limited. The remaining authors declare that the research was conducted in the absence of any commercial or financial relationships that could be construed as a potential conflict of interest.
